# P-1020. Randomized control trial to study the effectiveness of tablet Fluconazole, capsule Itraconazole and tablet Terbinafine in superficial dermatophytosis

**DOI:** 10.1093/ofid/ofae631.1210

**Published:** 2025-01-29

**Authors:** Debdeep Mitra

**Affiliations:** Command Hospital Air Force Bangalore, Bangalore, Karnataka, India

## Abstract

**Background:**

According to the World Health Organization, dermatophytosis is a relatively frequent superficial fungal infection of the keratinizing structures of the body with a prevalence of 20% - 25% worldwide. The organism is more widespread in tropical areas, with India acting as the main infection hub, because it prefers a hot and humid climate. Tinea corporis is the most common kind of superficial dermatophytic infection, second most common is tinea cruris followed by tinea capitis, tinea unguium, tinea faciei, tinea manuum and tinea pedis. Hot, humid weather, overcrowding, poverty, poor cleanliness, lack of health-care facilities, immunosuppression, and irrational drug use are all risk factors for dermatophytosis.

Fungal skin infection responding to Oral Itraconazole

**Figure:**
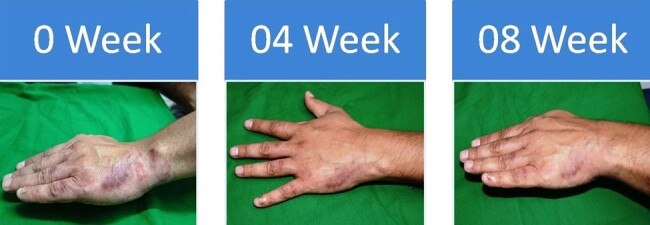

Clinical response of Dermatophyte infection to Oral Itraconazole at 4 and 8 weeks as compared to baseline

**Methods:**

Total of 180 patients were included in the study and divided into 3 groups based on the day of visit to the hospital and antifungals Fluconazolee, itraconazole or Terbinafine were randomly prescribed to them (60 patients in each group). Patients were followed up at 4th and 8th week for therapy response.

**Figure:**
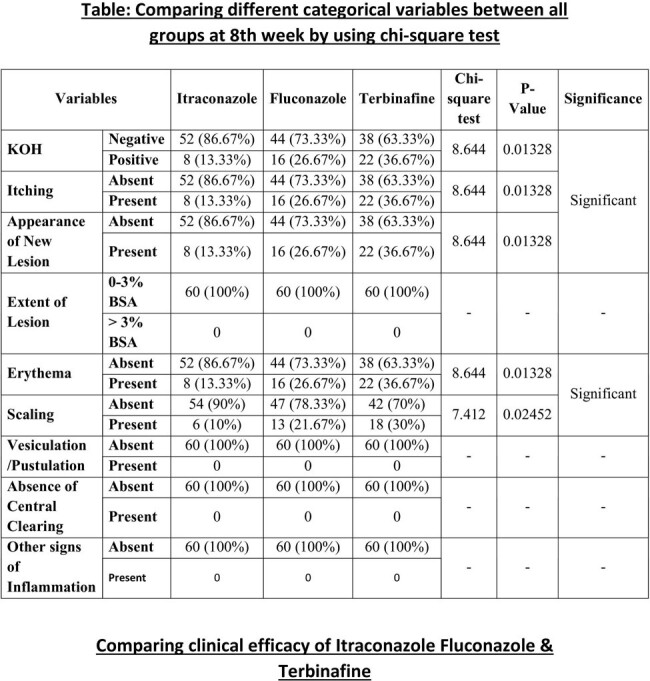

**Results:**

At the end of 8th week KOH (Potassium hydroxide) mount was found to be negative in 86.67%, 73.33% and 63.33% among Itraconazole Fluconazole and Terbinafine groups respectively which was highly significant.

**Conclusion:**

Dermatophytosis is comparatively more commonly seen in physically active age group. Clinical features waned off quicker and better with Itraconazole in comparison to Fluconazole and Terbinafine. Again fluconazole was found to be yielding better clinical outcome than Terbinafine. Overall outcome of patients with dermatophytosis was found to be more satisfactory with a 4 week oral dose of itraconazole. So from this study we conclude that Itraconazole is a better choice of drug for Dermatophytosis compared to Fluconazole and Terbinafine. Also fluconazole is comparatively a better choice than Terbinafine for the same.

**Disclosures:**

**All Authors**: No reported disclosures

